# Novel Hybrid Adsorption-Electrodialysis (AdED) System for Removal of Boron from Geothermal Brine

**DOI:** 10.1021/acsomega.2c06046

**Published:** 2022-12-01

**Authors:** Bekir Fırat Altınbaş, Ceren Orak, Hatice Eser Ökten, Aslı Yüksel

**Affiliations:** † Department of Chemical Engineering, Izmir Institute of Technology, Urla35430, Izmir, Turkey; ‡ Department of Environmental Engineering, Izmir Institute of Technology, Urla35430, Izmir, Turkey; § Geothermal Energy Research and Application Center, Izmir Institute of Technology, Urla35430, Izmir, Turkey

## Abstract

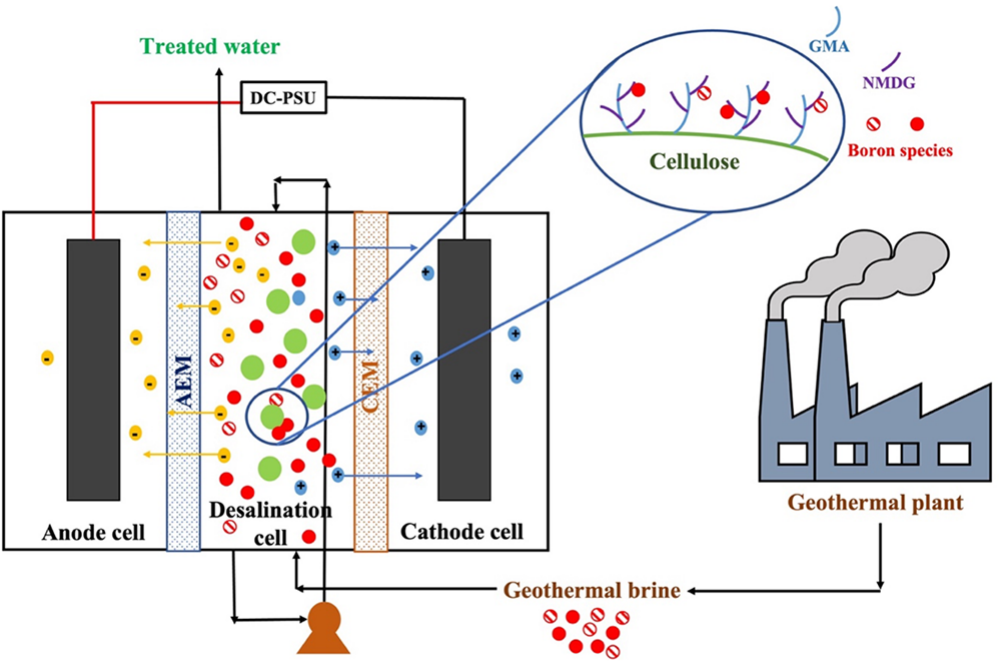

A novel hybrid adsorption-electrodialysis (AdED) system to remove environmentally harmful boron from geothermal brine was designed and effective operating parameters such as pH, voltage, and flow rate were studied. A cellulose-based adsorbent was synthesized from glycidyl methacrylate (GMA) grafted cellulose and modified with a boron selective n-methyl-d-glucamine (NMDG) group and characterized with SEM-EDX, FT-IR, and TGA analyses. Batch adsorption studies revealed that cellulose-based adsorbent showed a remarkable boron removal capacity (19.29 mg/g), a wide stable operating pH range (2–10), and an adsorption process that followed the Freundlich isotherm (*R*^2^ = 0.95) and pseudo-second-order kinetics (*R*^2^ = 0.99). In the hybrid AdED system, the optimum operating parameters for boron removal were found to be a pH of 10, a voltage of 10 V, a flow rate of 100 mL/min, and an adsorbent dosage of 4 g/L. The presence of the adsorbent in the hybrid system increased boron removal from real geothermal brine (containing 199 ppm boron) from 7.2% to 73.3%. The results indicate that the designed AdED system performs better than bare electrodialysis for boron removal from ion-rich real geothermal brine while utilizing environmentally friendly cellulose-based adsorbent.

## 1 Introduction

Boron compounds, while being essential for life, are also used widely in industries such as glass, detergents, fertilizers, electronics, cosmetics, pharmaceuticals, fuels, nuclear and catalysts industries, and so on, with the biggest share coming from the glass industry.^1,2^ The presence of boron in many industries produces boron-contaminated water and boron’s presence in geothermal brine results in the accumulation of boron in surface water after being processed from geothermal plants.^3^ Due to the toxic nature of excess boron for living beings and the narrow allowable range of boron in plants, in 2011, the World Health Organization (WHO) stated that the maximum amount of boron permitted in drinking water is 2.4 mg/L, and in irrigation water, the limitation was set to 1.0 mg/L.^4^ Therefore, it is important to remove boron from brines and spent solutions. There are various methods for the removal of boron, and these methods can be applied depending on the concentration of boron and the nature of the medium, both of which affect the present boron species in the medium, dictating their charge and size. The methods including evaporation, crystallization, or extraction are commonly used more for production of boric acid compounds than to remove boron in wastewater.^5^ The other methods for removal include reverse osmosis, ion exchange, nanofiltration, adsorption, chemical coagulation, and electrocoagulation. Membrane processes and adsorption are considered to be viable methods for removing pollutants from contaminated water due to ease of operation and separation, and these result in less contamination after operation.^6−8^ These two methods could be considered as a promising solution for boron removal.^9^ In addition to these boron removal methods, hybrid systems such as micelle enhanced ultrafiltration (MEUF), polymer enhanced ultrafiltration (PEUF), or adsorption membrane filtration (AMF) could be used and gained interest for boron removal from various water sources.^5,10−12^ For instance, Kabay et al. described a hybrid system that consists of adsorption and membrane filtration for boron removal from seawater reverse osmosis permeate.^13^

**Figure 1:**
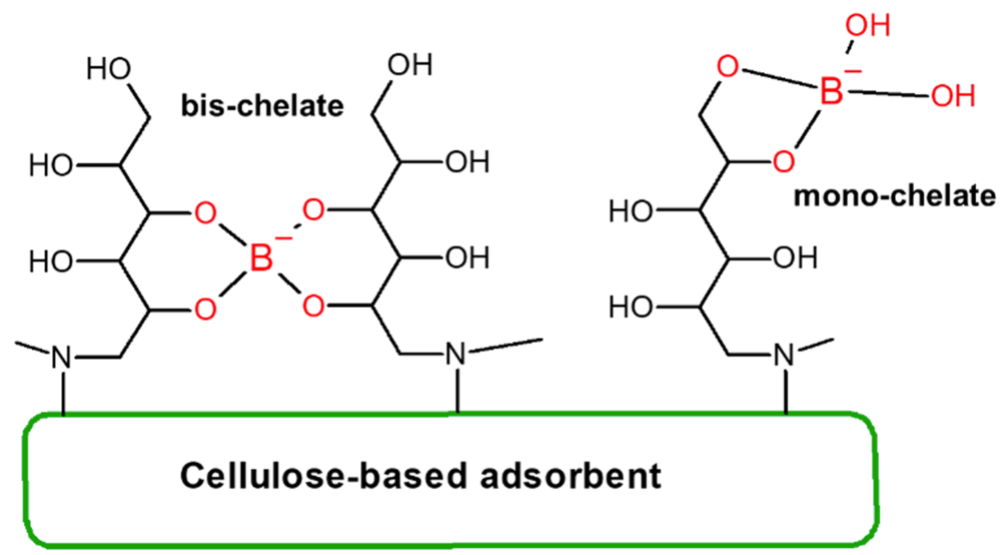
Boron removal mechanism of the NMDG functional group.

In this context, a novel hybrid adsorption electrodialysis water treatment technology (AdED) that removes charged borate ions (with electrodialysis and adsorption) and undissociated boric acid (H_3_BO_3_) with adsorption from geothermal brine was developed. For the hybrid system, a novel and environmentally friendly cellulose-based adsorbent containing glycidyl methacrylate (GMA) and a methyl-d-glucamine (NMDG) functional group was designed. Commercial boron selective adsorbents are mostly produced from a synthetic support such as polystyrene, and therefore utilization of low-cost and environmentally friendly cellulose is a viable option for sustainability.^7,14−16^ Graft polymerization of GMA with benzoyl peroxide as initiator onto cellulose was employed since GMA provides highly polymerizable vinyl groups and epoxy rings that can host many functional groups including NMDG.^17^ The NMDG functional group provides selective removal of the boron species present in solutions by forming monochelates and bis-chelates as shown in Figure 1; therefore, it is a good candidate for boron removal.^5,9,18−21^

After the cellulose-based adsorbent was synthesized, a characterization study comprising SEM, FT-IR, and TGA analyses was performed. Adsorption parameters for the synthesized adsorbent such as pH, adsorbent dosage, and concentration effects were investigated, and adsorption isotherms and kinetic models were studied to describe the adsorption process. The hybrid system was then tested for boron removal from the boron model solution and real geothermal brine. In this context, the effects of operating parameters such as pH, voltage, and flow rate were investigated. Hybrid system was tested with and without the adsorbent, and optimum operating parameters for boron removal were determined.

## 2 Results and Discussion

### 2.1 Characterization Study

The microcrystalline cellulose was grafted with GMA to attach boron selective NMDG groups to the resulting epoxy rings on the grafted cellulose. GMA was grafted onto the surface of cellulose with benzoyl peroxide as initiator for the reaction. Grafting efficiency and percent grafting were calculated from the following equations:
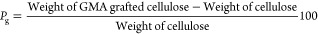
1
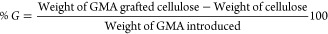
2It was found that *P*_g_ was 174.6% and %*G* was 63%. Comparing the results with the literature, higher grafting efficiency and percent grafting were achieved for the cellulose.^22^

After the NMDG binding reaction, the density of NMDG groups present on the adsorbent can be calculated from the weight of the remaining cellulose-based adsorbent and calculated from the following equation:
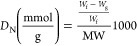
3where *W*_f_ is the weight of the cellulose-based adsorbent, *W*_g_ is the weight of the GMA-grafted cellulose, and MW = 195.2 g/mol for NMDG. *D*_N_ was found to be 2.1 mmol/g, which was similar to other NMDG type adsorbents reported in the literature that were prepared with various solvents (i.e., water, dioxane).^23−25^

**Figure 2:**
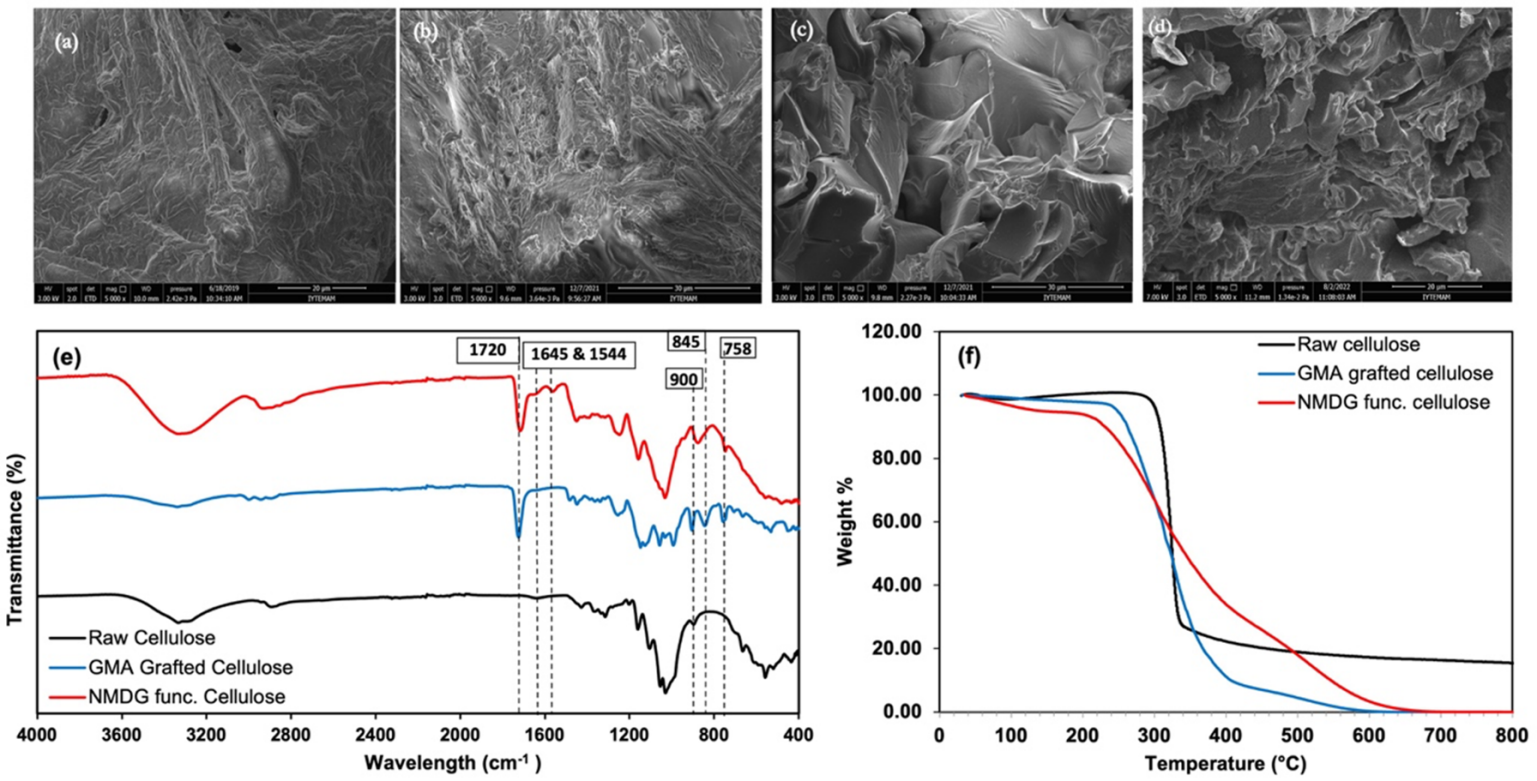
Characterization studies: SEM images (with magnification of 5000×) of (a) raw cellulose, (b) grafted cellulose, (c) NMDG functionalized cellulose, and (d) used functionalized adsorbent. (e) FT-IR spectra of raw cellulose, GMA grafted cellulose, and NMDG functionalized cellulose. (f) TGA thermograms of raw cellulose, GMA grafted cellulose, and NMDG functionalized cellulose.

In the context of characterization study, SEM, FT-IR, and TGA analyses were performed to the synthesized adsorbent and the results were given in Figure 2.

The morphological structure of the grafted cellulose surface became rougher than raw cellulose due to the addition of GMA into its structure. It is clearly seen that NMDG-functionalized cellulose turns into a more crystalline, smooth structure, while raw cellulose and grafted cellulose have more fibrous structures. Therefore, the surface of the raw cellulose has undergone morphological changes because of grafting with GMA and introduction of NMDG functional groups into its structure.

In Figure 2e, when FT-IR spectra of cellulose and grafted cellulose are compared, due to the GMA introduction into the structure of raw cellulose, the characteristic peak of C=O related to GMA formed with the addition of a high amount of carboxyl groups at 1722 cm^–1^ and the peaks attributed to the epoxy rings at 845, 757–788, and 905 cm^–1^ formed, and the results are in line with the literature.^17,25,26^ Also, the addition of GMA to the −OH groups of cellulose as proposed in the reaction scheme given in Figure 9, resulted in decrease of the −OH peaks at 3320 cm^–1^ and suggesting successful binding of GMA to raw cellulose.^23^ As a result of NMDG binding to grafted cellulose, previously disappeared −OH peaks at 3320 cm^–1^ reappeared due to the addition of −OH groups present in the NMDG structure. The peaks belonging to amide 1 and amide 2 groups were observed at 1645 and 1544 cm^–1^, respectively.^23,27^ Since the only nitrogen source was the NMDG group, the results also proved that functional group binding was successfully accomplished. In addition, the peaks belonging to the epoxy groups that were present in grafted cellulose disappeared or decreased with the binding of the NMDG functional group, thus suggesting the NMDG groups were attached to epoxy rings as shown in the reaction scheme, and this phenomenon is in line with the literature.^16,22,26^

Thermograms of raw cellulose, grafted cellulose, and NMDG-functionalized cellulose are given in Figure 2f. Depending on the individual pyrolysis processes, similar thermal characteristics were observed for raw cellulose and grafted cellulose. In addition, the loss of mass around 100 °C in all three materials is attributed to the moisture that may be in the samples, indicating the loss of water molecules adsorbed and bound by the samples. In raw cellulose, mass loss between 300 and 350 °C indicates decomposition of the cellulose.^28^ The grafted cellulose started to decompose at higher temperatures compared to raw cellulose and the mass loss continued at higher temperatures. It can be deduced that the presence of GMA groups in the grafted cellulose led that the decomposition of grafted cellulose started at higher temperatures. Additionally, it was observed that the thermal decomposition characteristic of NMDG functionalized cellulose was different. Each modification has changed the decomposition temperature for samples. According to the thermograms, the synthesized adsorbent showed thermal stability up to temperatures of about 300 °C.^29^

Consequently, the results of characterization study (SEM, FT-IR, and TGA) of cellulose-based adsorbent showed that the adsorbent was successfully synthesized.

### 2.2 Batch Adsorption Studies

#### 2.2.1 Effect of Adsorbent Dosage and pH

**Figure 3:**
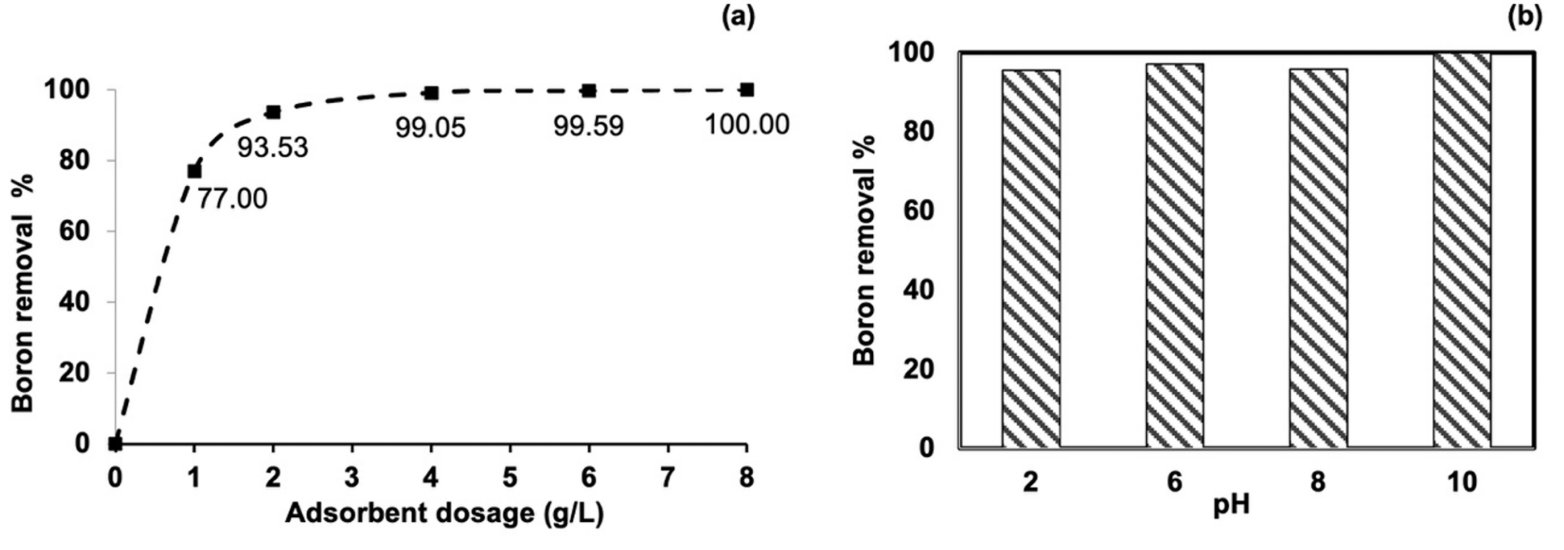
Effect of parameters: (a) Cellulose-based adsorbent amount (conditions: *C*_0_ = 10 ppm, *T* = 25 °C, pH 10, adsorbent dosage = 1–8 g/L). (b) Effect of pH (conditions: *C*_0_ = 10 ppm, *T* = 25 °C, adsorbent dosage = 4 g/L pH 2–10).

Batch adsorption studies were performed using 10 ppm boron model solution at various pH and adsorbent dosage levels for 24 h to determine the optimum adsorbent dosage and operating pH to employ in the AdED system for boron removal and the results were given in Figure 3a. Almost complete boron removal (99.05%) was observed at 4 g/L adsorbent dosage. Increasing adsorbent dosage further did not increase the boron removal significantly. Hence, 4 g/L adsorbent dosage was selected as the optimum for this adsorption conditions. After selecting optimum adsorbent dosage, the effect of pH over boron removal was investigated in the range of pH 2 to 10 for 24 h using 10 ppm boron model solution.

In aqueous solutions, boron can be present as boric acid with a p*K*_a_ value of 9.2 and borate ions that are negatively charged. When the solution pH increases, the resulting −OH ions can compete with borates on the adsorbent surface and also can interfere with the process of electrodialysis in the hybrid system. The results of the pH effect over boron removal are given in Figure 3b.

In Figure 3b, the highest boron removal was observed at pH 10. Cellulose-based adsorbent showed high stability for boron removal in a wide pH range and similar results are reported for NMDG-functionalized adsorbents in the literature.^28−30^ Consequently, to also support boron removal in the electrodialysis system by generating borate ions, the optimum pH was selected as 10 in this study. The effect of pH on the hybrid system is discussed in detail in Section 2.3.1.

#### 2.2.2 Adsorption Kinetics of Cellulose-Based Adsorbent

**Figure 4:**
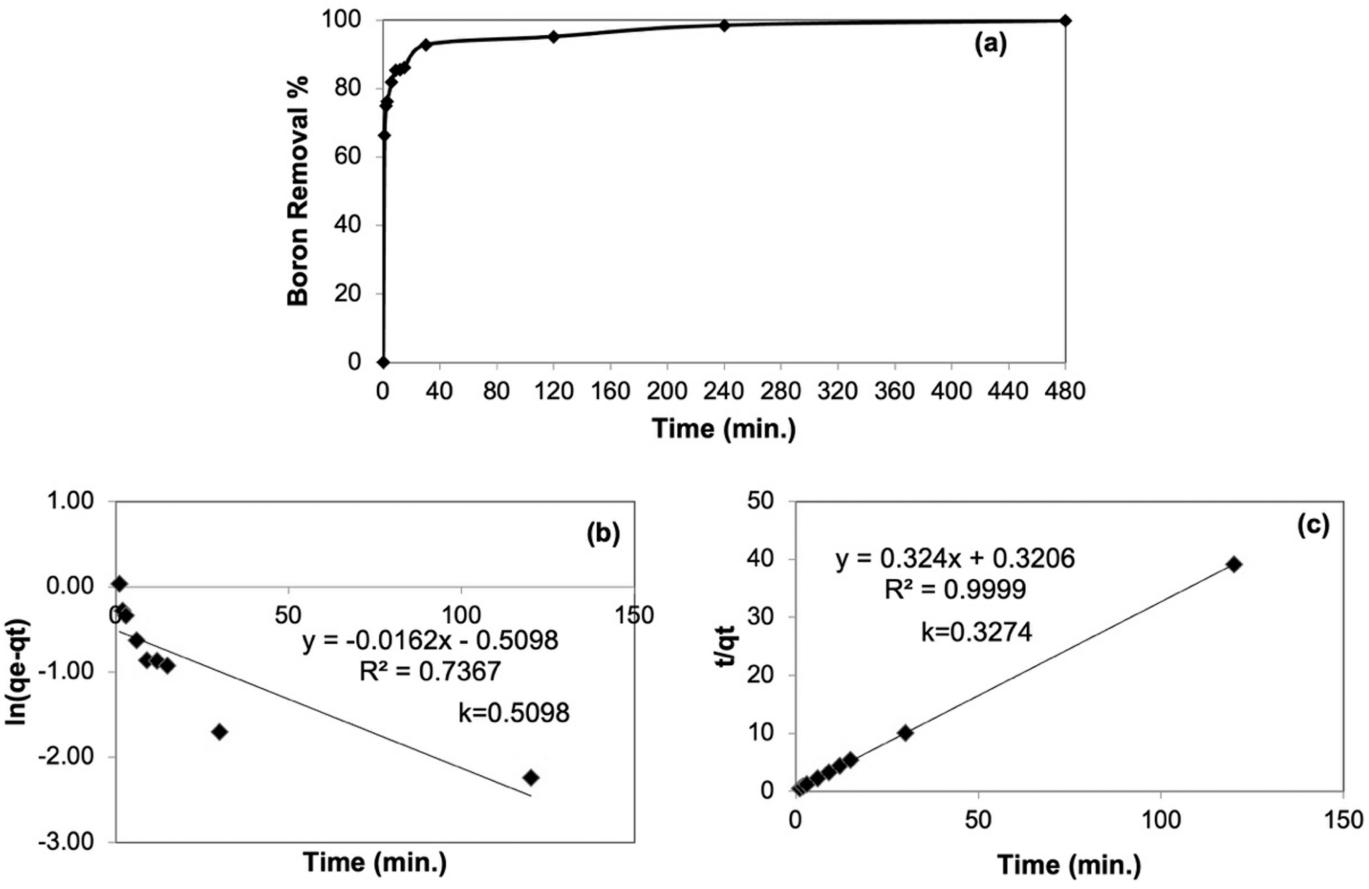
Kinetic study: (a) Effect of contact time on boron removal % (conditions: *C*_0_ = 10 ppm, *T* = 25 °C, pH 10, adsorbent dosage = 4 g/L). (b) Pseudo-first-order kinetic model for boron removal (conditions: *C*_0_ = 10 ppm, *T* = 25 °C, pH 10, adsorbent dosage = 4 g/L). (c) Pseudo-second-order kinetic model for boron removal (adsorption conditions: *C*_0_ = 10 ppm, *T* = 25 °C, pH 10, adsorbent dosage = 4 g/L).

To understand the kinetic behavior of the synthesized adsorbent, we carried out a kinetic study with 10 ppm boron model solution using the optimum adsorbent amount (4 g/L) at ambient temperature for 24 h and kinetic study results were given in Figure 4a. After 4 h, equilibrium was reached. Boron uptake of the adsorbent in the first minute was found as 66.2%; therefore, the fast kinetic nature of the adsorbent was observed. Linearized forms of the pseudo-first-order kinetic model (eq 4) and the pseudo-second-order kinetic model (eq 5) were applied to experimental data.^31^
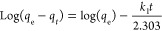
4
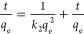
5where *q*_e_ (mg/g) is the boron adsorbed in equilibrium and *q*_t_ (mg/g) is the boron adsorbed at a certain time, while *t* is time (min^–1^) with *k* the rate constants for the models. The results of the pseudo-first-order model and the pseudo-second-order model were given in Figure 4b and Figure 4c, respectively. As seen in Figure 4b, c, the *R*^2^ value for the pseudo-second-order model was greater than that for the pseudo-first-order model, thus suggesting the adsorption kinetic mechanism followed the pseudo-second-order model. Several other boron selective adsorbents in the literature containing the NMDG functional group also followed pseudo-second-order model kinetics.^22,28,30,32^

#### 2.2.3 Adsorption Isotherms of Cellulose-Based Adsorbent

In order to determine the interaction of the cellulose-based adsorbent and the boron species, two most common adsorption isotherms were applied to the experimental data. Experimental data was fitted to the models using least-squares regression method and the R^2^ values of models were compared to find the best fitting model.^33^

Langmuir isotherm defines adsorption by a single-layer homogeneous adsorption on surface while Freundlich isotherm assumes a multilayer heterogeneous adsorption present on the surface.^6,34^ The calculated adsorption parameters were given in Table 1. For cellulose-based adsorbent, Freundlich isotherm with a R^2^ value of 0.949 fits the experimental data better than Langmuir isotherm. The result suggests a multilayer heterogeneous adsorption of boron species onto NMDG functional group.^21^ Maximum adsorption capacity of the adsorbent was calculated from Langmuir adsorption isotherm and was found as 19.29 mg/g.

**Table 1:** Langmuir and Freundlich Adsorption Isotherm Model Parameters of N-OPW

Langmuir adsorption isotherm parameters				Freundlich adsorption isotherm parameters			
*K*_L_	*Q*_max_(mg/g)	*R*^2^ (nonlinear, Qe vs Ce plot)	*R*^2^(linear, *C*_e_/*Q*_e_ vs *C*_e_ plot)	*n*	*K*_F_	*R*^2^ (nonlinear, *Q*_e_ vs *C*_e_ plot)	*R*^2^ (linear, ln *Q*_e_ vs ln *C*_e_ plot)
0.12	19.29	0.910	0.886	3.16	4.01	0.949	0.845

Adsorption capacity was compared with some other NMDG-functionalized adsorbents that were reported in the literature and the studies were given in Table 2. The adsorption capacity of the synthesized cellulose-based adsorbent was remarkably higher than most of the NMDG type adsorbents reported in the literature.

**Table 2:** Comparison of Adsorption Capacities of Different NMDG Functionalized Adsorbents

adsorbent	adsorption capacity(mg/g)	ref
nylon-6-VBC-NMDG	13.8	(4)
silica-polyamine-NMDG	16.76	(21)
Diaion CRB02 boron-selective resin (commercial)	13.18	(30)
Diaion CRB05 boron-selective resin (commercial)	17.45	(30)
PE-GMA-NMDG	14.5	(24)
Si-NMDG	21.84	(35)
cellulose-GMA-NMG	4.71	(30)
cellulose-based adsorbent	19.29	this study

### 2.3 Boron Removal by a Hybrid (AdED) System

In a hybrid system, before the addition of cellulose-based adsorbent, the effects of pH, flow rate and voltage over boron removal percentage were investigated to find the optimum conditions for boron removal from the boron model solution. After finding the optimum parameters, hybrid system was operated with cellulose-based adsorbent for removal of boron from both the boron model solution and geothermal brine. Geothermal brine was analyzed via ICP-OES (Agilent Technologies, 5110) and IC (Thermo Scientific Dionex ICS-5000) to identify the anions and cations and to determine their concentrations and the results were given in Table 3.

**Table 3:** Characterization of Geothermal Brine

anions	concentration (ppm)	cations	concentration (ppm)
fluorine	9.13	lithium	5.69
chlorine	249.9	boron	119.05
nitrite		potassium	38.08
bromine	1.16	magnesium	0.38
nitrate	0.39	calcium	3.55
sulfate	14.75	arsenic	1.59
phosphate	0.17	sodium	982

#### 2.3.1 Effect of pH, Voltage, and Flow Rate

**Figure 5:**
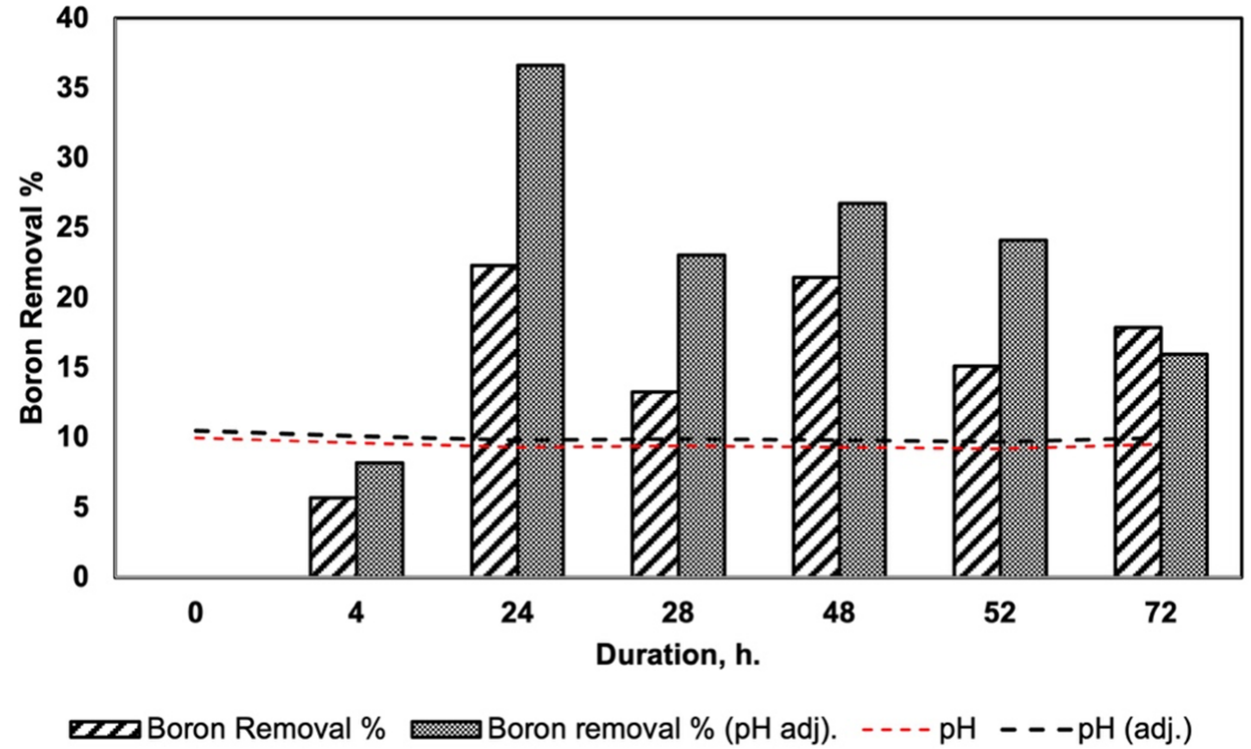
Effect of pH over boron removal (conditions: pH 10, [Boron]_0_ = 10 ppm, flow rate = 100 mL/min, *V* = 0 V).

First, the effect of pH was examined using the boron model solution over 72 h. Boron removal was carried out without applying any voltage on the system and the pH of the model solution was adjusted to 9.5, which is above the p*K*_a_ value of boric acid (9.2), to increase borate ions dominance, hence increasing the migration to the anion cell. However, as seen in Figure 5, with time, the pH of the desalination cell decreased. The pH of the boron solution decreased to less than 9.5 and even 9.2 depending on the time during the experiment. When the pH decreases, uncharged boron ions stopped migration and chargeless boron species came back to the desalination cell. To keep the pH value at 9.5, we used NaOH solution in the following experiments. After that, the pH of boron solution was adjusted while the boron removal % was investigated at pH 10 as constant. The changes in boron concentration in the desalination cell were given in Figure 5, and the results showed that the boron ions that moved to the anion cell from desalination cell did not turn back when the pH of desalination cell was kept constant. Batch adsorption studies of boron also showed better performance at pH 10. Therefore, the further experiments were carried out at pH 10 for desalination cell.

**Figure 6:**
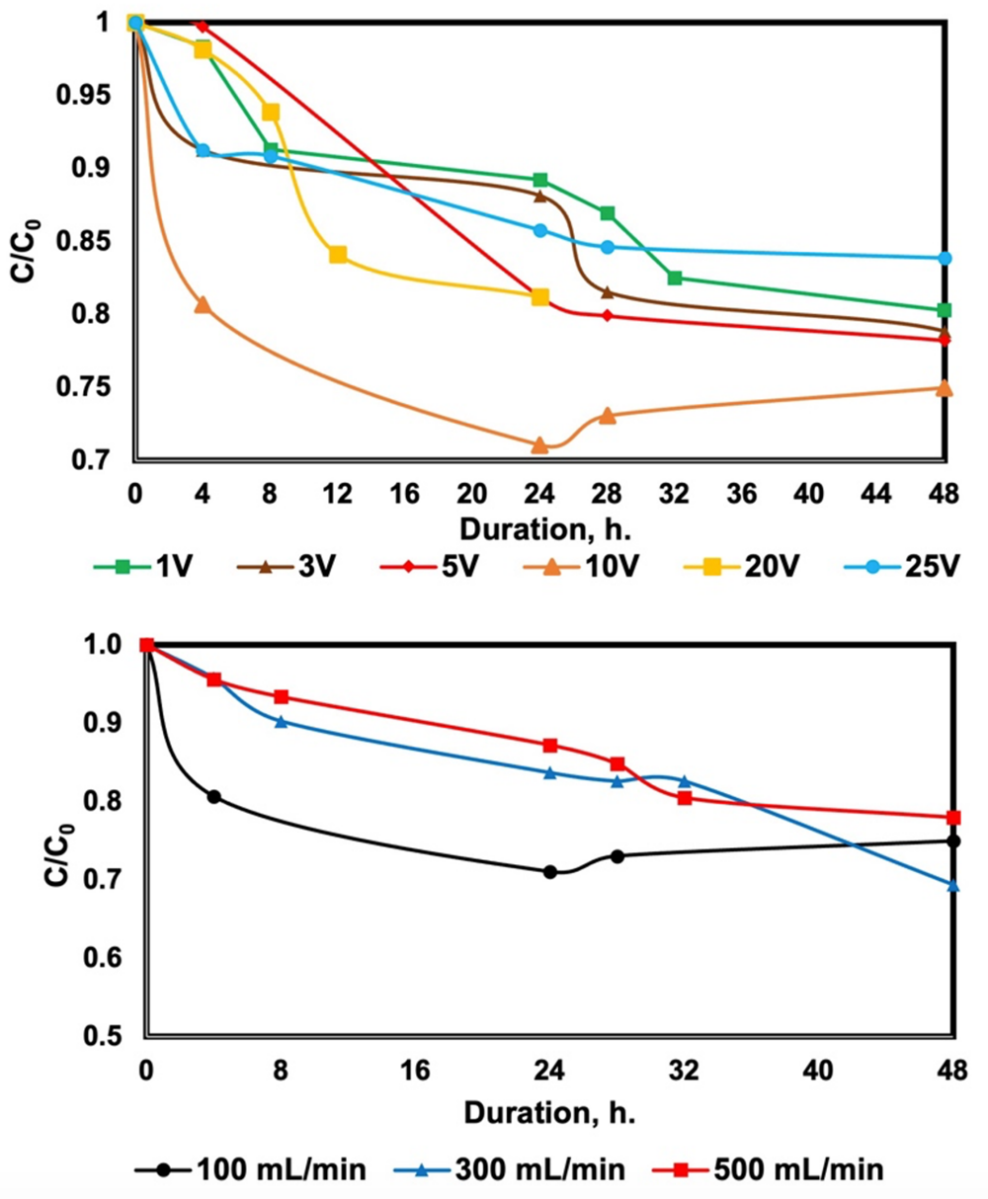
(a) Effect of voltage over boron removal (conditions: pH 10, [Boron]_0_ = 10 ppm, flow rate = 100 mL/min, *V* = 1, 3, 5, 10, 20, and 25 V). (b) Effect of flow rate (conditions: pH 10, [Boron]_0_ = 10 ppm, *V* = 10 V, flow rate = 100, 300, and 500 mL/min).

The impact of voltage over boron removal was examined by applying different voltages (1, 3, 5, 10, 20, and 25 V). The obtained results were given in Figure 6a. First, considering both pH and voltage effect results, the boron removal % was highest at 24 h so that the experimental duration was kept as 24 h. The highest boron removal % was achieved applying 10 V at the end of 24 h. Increasing the voltage yielded better results up to 10 V and had a decrease on boron removal when voltage was further increased. A similar phenomenon was discussed in the literature and the possible reason is the limiting current density.^36,37^ When the number of ions in the desalination cell is not enough to bear the current, cell resistance increases, resulting in a water splitting reaction that reduces the energy for the electrodialysis system, resulting in lower efficiencies.^38,39^ Therefore, 10 V was chosen as the optimum voltage for this system.

After selecting the optimum pH and voltage for the system, the effect of the flow rate over boron removal % was investigated. In this context, flow rates of 100, 300, and 500 mL/min were tested, and the results were given in Figure 6b. Based on the results, highest boron removal % was observed at the lowest flow rate and hence, optimum flow rate was selected as 100 mL/min.

#### 2.3.2 Boron Removal from Geothermal Brine

**Figure 7:**
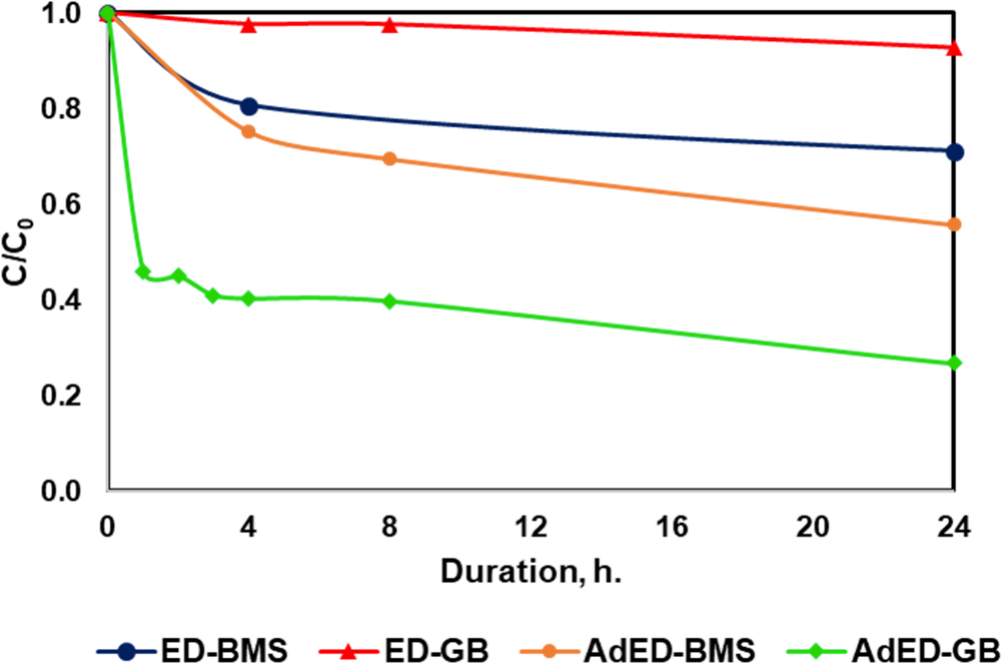
Effect of adsorbent usage in the AdED system (conditions: pH 10, [Boron]_0_ = 10 ppm for model solution, [Boron]_0_ = 119 ppm for geothermal brine, *V* = 10 V, flow rate = 100 mL/min). *ED, electrodialysis; GB, geothermal brine; AdED, adsorption+electrodialysis (hybrid); BMS, boron model solution.

Lastly, the cellulose-based adsorbent was introduced into the desalination cell to investigate the effect of adsorbent usage over boron removal from the boron model solution (BMS) and real geothermal brine in hybrid system. Characterization of geothermal brine was given in Table 1. and obtained results from the experiments were given in Figure 7. Without the adsorbent, geothermal brine had 7.2% of boron removal after the operation, indicating that ions present in brine interfere with the electrodialysis process. After the cellulose-based adsorbent was introduced to the system, boron removal increased to 73.3% from 7.2% for geothermal brine and to 44.3% from 30% for the boron model solution. The addition of adsorbent caused a considerable impact over boron removal % for both solutions. Despite the high ion content of the geothermal brine, boron removal enhanced in the hybrid system. In literature, presence of ions in the boron containing solution increased the boron removal using NMDG functionalized adsorbents,^29^ so the enhancement of adsorption with presence of ions was also observed in this study. Also, the higher concentration gradient of geothermal brine (119 ppm versus 10 ppm) would increase the boron adsorption compared to the boron model solution.

Guesmi et al. studied over boron removal from water that contains 5 ppm boron via electrodialysis, and the highest efficiency was 43.5% for pH 10, 100 L/h of flow rate and 25 min of reaction duration.^40^ Turek et al. studied over boron removal from seawater reverse osmosis permeate using two model solutions that contain 2.25 and 1.3 ppm boron via electrodialysis. In this study, AMX and CMX Neosepta (Tokuyama Co.) membranes were used, and the boron concentration decreased to 0.4 ppm for both model solutions.^41^ Yazicigil and Oztekin studied over electrodialysis of boron using 0.1 M boric acid solution. In this study, anion exchange membrane was used to separate anion and cation cells. Applying 45 mA, almost 0.4 mmol boron moved through anion exchange membrane (AHA).^42^ Melnik et al. investigated boron behavior throughout the desalination of sea and underground water via electrodialysis and in this study boron removal % changed between 30 and 89 at different working conditions. The highest boron removal was achieved using MK-40 and MA-40 membranes and initial boron concentration was 4.7 ppm at pH of 10.9. However, in each trial, the final boron concentration was not below 0.4 ppm so that it could be concluded that sorbents could be used to enhance boron removal.^43^ In the present study, the efficiency of electrodialysis was enhanced using cellulose-based adsorbent in the hybrid water treatment process; 44.3% of boron removal was achieved from 10 ppm model solution and 73.3% for real geothermal brine. Compared to the literature, higher boron concentrations were studied in the present study and relatively higher efficiencies of boron removal from geothermal water were obtained in the hybrid system.

## 3 Conclusion

A novel hybrid electrodialysis-adsorption (AdED) system containing a cellulose-based adsorbent was designed to remove boron from geothermal brine since it is harmful for the environment. Environmentally friendly cellulose-based adsorbent was successfully synthesized from GMA-grafted cellulose and modified with NMDG and based on the SEM-EDX, FT-IR, and TGA results, the synthesis of the adsorbent was successful. Based on results of batch adsorption study, the capacity of cellulose-based adsorbent was found as 19.29 mg/g in a wide and stable operation pH range (2–10). Adsorption process followed Freundlich isotherm and (*R*^2^ = 0.95) and pseudo-second-order kinetics (*R*^2^ = 0.99). In hybrid AdED system, the boron removal experiments were carried out in the absence and presence of the cellulose-based boron selective adsorbent and optimum operating parameters were found as pH of 10, voltage of 10 V, flow rate of 100 mL/min and, adsorbent dosage of 4 g/L. In the absence of the boron selective adsorbent, low boron removal (7.2%) from geothermal brine was observed, likely due to competing ions present in the brine and thus reducing the efficiency of the electrodialysis process. However, the addition of boron selective cellulose-based adsorbent into the desalination cell increased the boron removal from 7.2 to 73.3% from geothermal brine, indicating the hybrid system can enhance boron removal in geothermal brines. Therefore, this study showed that environmentally friendly cellulose can be used in a novel AdED system to enhance boron removal from geothermal brine.

## 4 Materials and Methods

### 4.1 Materials and Chemicals

The hybrid AdED system was tailored from plexiglass. The anion exchange membrane (AEM, AMI- 7001, Membrane International Inc., USA) and the cation exchange membrane (CEM, CMI-7000, Membrane International Inc., USA) were supplied to construct a hybrid (electrodialysis and adsorption) system for boron removal from geothermal water streams. Real geothermal brine was supplied from Alkan Geothermal Power Plant, Turkey. For the synthesis of cellulose-based boron selective adsorbents, we purchased microcrystalline cellulose powder from Sigma-Aldrich. Glycidyl methacrylate (GMA, 97%), *N*-methyl-d-glucamine (NMDG, 99%), and benzoyl peroxide (75%) were purchased from Acros Organics. Additionally, acetone (99.8%) and boric acid (99.5%), which were used to prepare the stock boron solution (1000 ppm), were supplied from Merck.

### 4.2 Novel Hybrid Adsorption-Electrodialysis (AdED) System Setup

**Figure 8:**
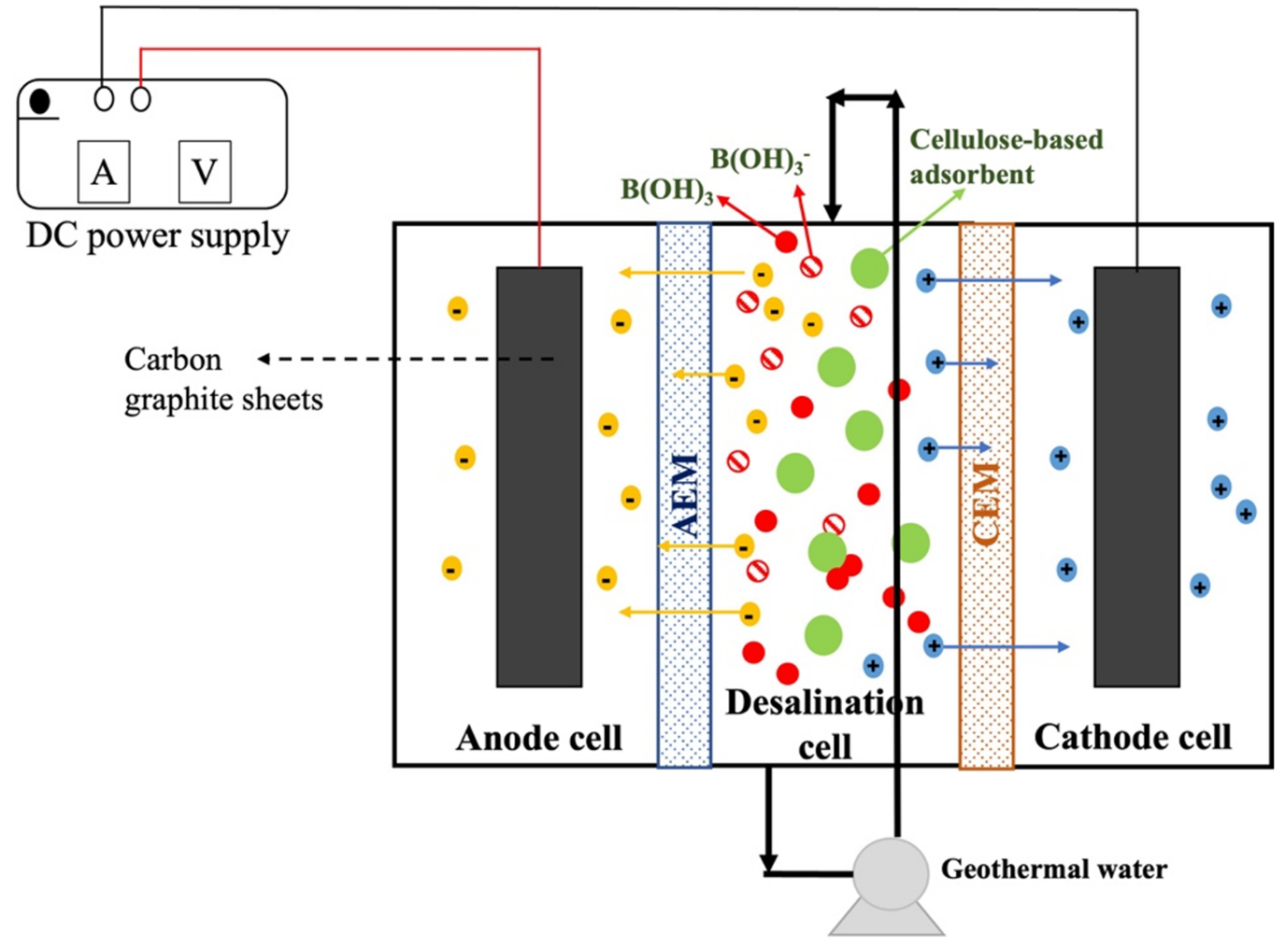
Novel hybrid adsorption-electrodialysis (AdED) system.

The hybrid system for boron removal consists of three compartments: an anode cell, a desalination cell, and a cathode cell. The hybrid system is illustrated in Figure 8. Each compartment has 3.5 L of volume. Anode and desalination cells were separated by an anion exchange membrane (AEM), while desalination and cathode cells were separated by a cation exchange membrane (CEM). Moreover, to increase the electrochemical gradient between compartments, we placed carbon graphite sheets into the anode and cathode cells and connected to a power supply through a wire. A pump was used to introduce flow and mixing in the desalination cell. The newly synthesized cellulose-based adsorbent was put into the desalination cell, which is located between anode and cathode cells and, desired voltage was applied to the system through the power supply. To comprehend the boron removal efficiency of the hybrid system, first, we introduced boron model solution (*C*_0_ = 10 ppm) into the desalination cell. Anode and cathode cells were filled with deionized water and run in electrodialysis mode to optimize the system parameters such as flow rate, voltage, and pH. After that, the experiments were carried out using real geothermal brine, while newly synthesized cellulose-based adsorbent was introduced into the desalination cell so that adsorption and electrodialysis occur simultaneously.

### 4.3 Preparation of Cellulose-Based Adsorbent

#### 4.3.1 Synthesis of GMA-Grafted Cellulose

**Figure 9:**
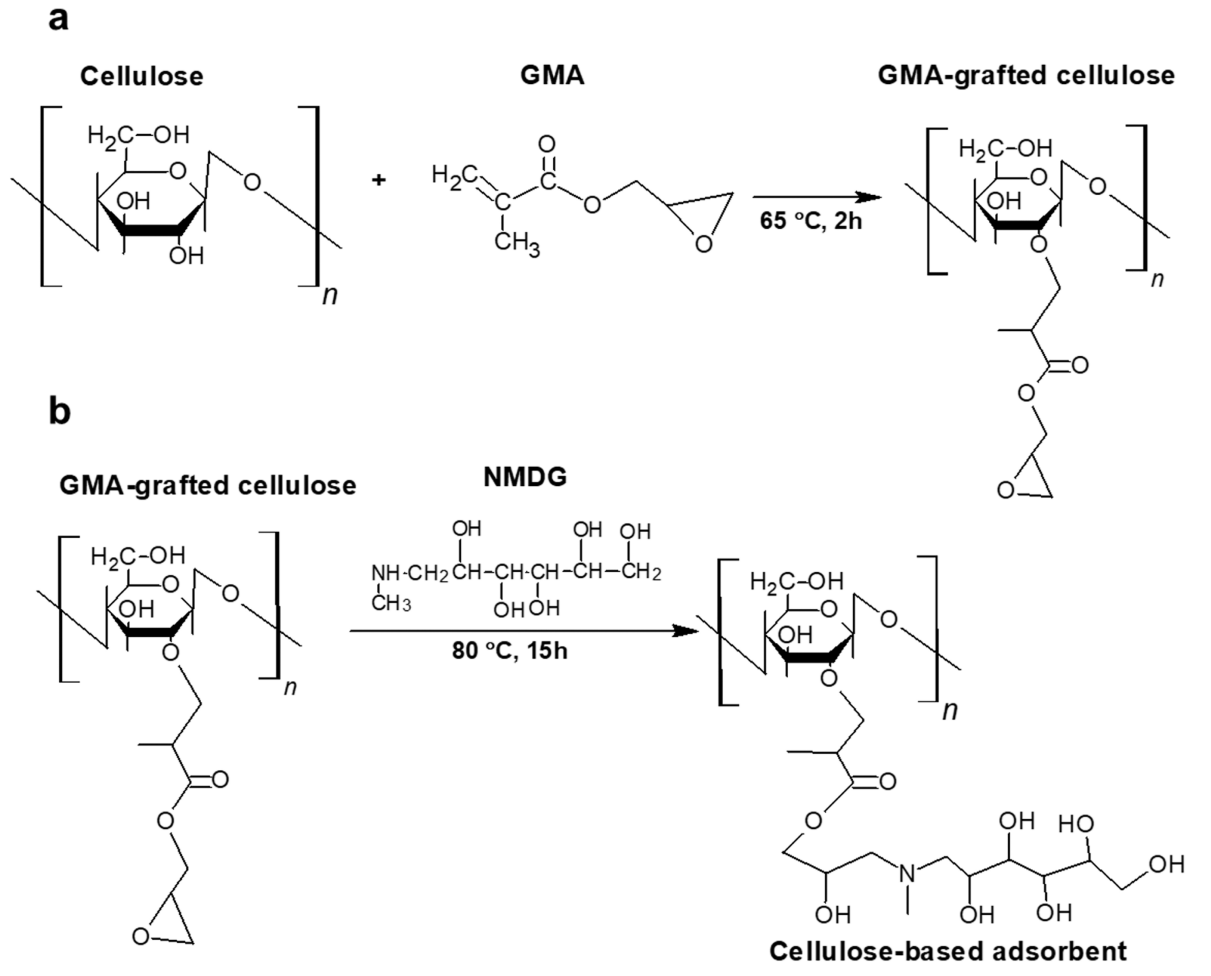
Reaction mechanism of (a) GMA grafting and (b) NMDG attachment.

To attach the NMDG functional group onto cellulose, we first grafted GMA onto cellulose. Three grams of microcrystalline cellulose, 8.2 g of glycidyl methacrylate, and 0.3 g of benzoyl peroxide as the initiator were mixed in deionized water (30 mL) and acetone (15 mL). The mixture was introduced to a three-neck flask and the air inside of the flask was purged using nitrogen gas. After the purging step, the mixture was heated to 65 °C and the polymerization reaction took place for 2 h. Figure 9a shows the reaction mechanism. The resulting mixture was washed with deionized water and acetone.

Since polyglycidyl methacrylate may have been produced in the reaction as well, 7 cycles of Soxhlet extraction were applied to remove polyglycidyl methacrylate homopolymer.^16,22,26^ The obtained GMA-grafted cellulose was dried at 50 °C overnight and then ground in a mortar.

#### 4.3.2 Functionalization of GMA-Grafted Cellulose

After the GMA-grafted cellulose was produced, to attach the NMDG functional group to epoxy rings of GMA, we reacted the GMA-grafted cellulose (3 g) with NMDG (16.1 g) in water (64 mL) for 15 h.^15^ The mixture was heated to 80 °C and constantly stirred throughout the reaction. Figure 9b shows the reaction mechanism. The resulting mixture was filtered and washed with excess water to remove the unreacted NMDG group, and then the remaining adsorbent was dried at 50 °C overnight.

### 4.4 Characterization of Cellulose-Based Adsorbent

The surface morphologies of cellulose, GMA-grafted cellulose, and cellulose-based adsorbent containing NMDG functional groups were analyzed via SEM (FEI QUANTA 250 FEG model) analysis. To observe the changes that occurred in the bond structures of raw cellulose, grafted cellulose, and NMDG functionalized cellulose, we recorded IR spectra in the range of 4000–400 cm^–1^ with a PerkinElmer UATR-FT-IR device at 4 cm^–1^ resolution and 20 scans per sample. Thermogravimetric analysis (Shimadzu, TGA-51) was used to determine the thermal stability of samples by heating them at 5 °C/min through nitrogen gas between 30 and 1000 °C.

### 4.5 Batch Adsorption Experiments for Cellulose-Based Adsorbent

To check the adsorption performance of the synthesized adsorbent before employing it in the hybrid system, we performed batch adsorption studies using 10 ppm boron model solution in 50 mL of closed plastic flasks in a shaking incubator at 180 rpm for 24 h. The dosage effect (0.05–0.4 g), pH effect (pH 2–10), concentration effect (5–130 ppm boron solution), and adsorption kinetics were examined. The pH adjustment of the boron model solution was performed using HCl and NaOH solutions. At the end of the experiments, the remaining liquid product was analyzed via ICP-OES to determine boron removal. The following equations were used to calculate the adsorption capacity (*q*) and boron removal (*r*):
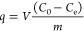
6
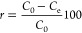
7where *q* is (mg/g), *V* is (L), *C* is (mg/L) and *m* is (g).

In order to describe the interaction of synthesized adsorbent and the boron species on the surface, adsorption isotherm models were used. Boron adsorption isotherms were modeled using experimental data with following Langmuir and Freundlich equations:
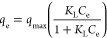
8
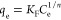
9where *q*_e_ is adsorption at equilibrium (mg/g), *q*_max_ is maximum boron adsorbed (mg/g), *K*_L_ is the Langmuir isotherm constant (L/mg), and *K*_F_ and *n* are Freundlich isotherm constants.^44^
